# Transcription by RNA polymerase III: insights into mechanism and regulation

**DOI:** 10.1042/BST20160062

**Published:** 2016-10-19

**Authors:** Tomasz W. Turowski, David Tollervey

**Affiliations:** Wellcome Trust Centre for Cell Biology, University of Edinburgh, Michael Swann Building, Kings Buildings, Mayfield Road, Edinburgh EH9 3JR, U.K.

**Keywords:** RNA polymerase III, transcription elongation, transcription factors, tRNA

## Abstract

The highly abundant, small stable RNAs that are synthesized by RNA polymerase III (RNAPIII) have key functional roles, particularly in the protein synthesis apparatus. Their expression is metabolically demanding, and is therefore coupled to changing demands for protein synthesis during cell growth and division. Here, we review the regulatory mechanisms that control the levels of RNAPIII transcripts and discuss their potential physiological relevance. Recent analyses have revealed differential regulation of tRNA expression at all steps on its biogenesis, with significant deregulation of mature tRNAs in cancer cells.

## Introduction

The basic machinery for RNA transcription evolved prior to the last common ancestor of all extant organisms. Bacteria and Archaea retain a single RNA polymerase (RNAP), whereas most Eukaryotes use three distinct polymerases for different classes of RNA, with partially overlapping sets of components [1]. Transcriptional regulation of protein-coding gene expression by RNA polymerase II (RNAPII) has been extensively analyzed. In contrast, regulation of rRNA transcription by RNAPI and tRNA transcription by RNAPIII is understood in considerably less mechanistic detail. These highly abundant RNAs are generally less responsive to regulation than short-lived mRNA species, but their expression is both a major metabolic activity and essential to underpin all cell growth and division. These components are therefore major features in the overall control of gene expression. The aim of this short review is to highlight recent work revealing variability in tRNA transcription, in the context of our current understanding of RNAPIII regulation.

## RNAPIII transcription initiation factors and sites

RNAPIII is specialized for transcription of short, abundant nonprotein-coding RNA transcripts. In addition to all tRNAs, RNAPIII transcribes the 5S rRNA and other essential RNAs, including the U6 small nuclear RNA (snRNA), the snR52 small nucleolar RNA and the RNA components of the signal recognition particle (*SRP1*) and RNase P (*RPR1*) [2].

The basic mechanisms of RNAPIII transcription have been studied in considerable detail. RNAPIII is a 17-subunit enzyme that functions together with 3 transcription factors: TFIIIA, TFIIIB and TFIIIC (reviewed in ref. [3]). TFIIIA is a single protein and is specific for transcription only of the 5S rRNA gene, *RDN5*. TFIIIB is composed of Brf1, Bdp1 and TBP (TATA-binding protein), which is common to all eukaryotic RNAPs. TFIIIC is a large, flexible six-subunit complex that recognizes the promoter elements of all RNAPIII transcription units [4]. In contrast to RNAPI and RNAPII, the promoter elements of most RNAPIII genes, including tRNAs and 5S rRNA, lie within the transcribed region. tRNA genes have two conserved internal elements, termed the A box and B box. These coincide with conserved structural features in the mature tRNA body, named the D loop and T loop, respectively. In the case of *RDN5*, the internal promoter elements consist of an A box plus a gene-specific C box that is recognized by the specific transcription factor, TFIIIA, which has no other essential functions [5] and mediates TFIIIC binding. Other RNAPIII transcripts show slightly different organization; for example, the internal promoter elements at the 5′-end of the RNase P RNA are cleaved off from the mature RNA. In contrast, the U6 snRNA has an internal A box, a B box located downstream of the termination signal and an upstream TATA box that is bound by TBP [6].

## RNAPIII initiation and elongation

**Figure 1. BST-2016-0062F1:**
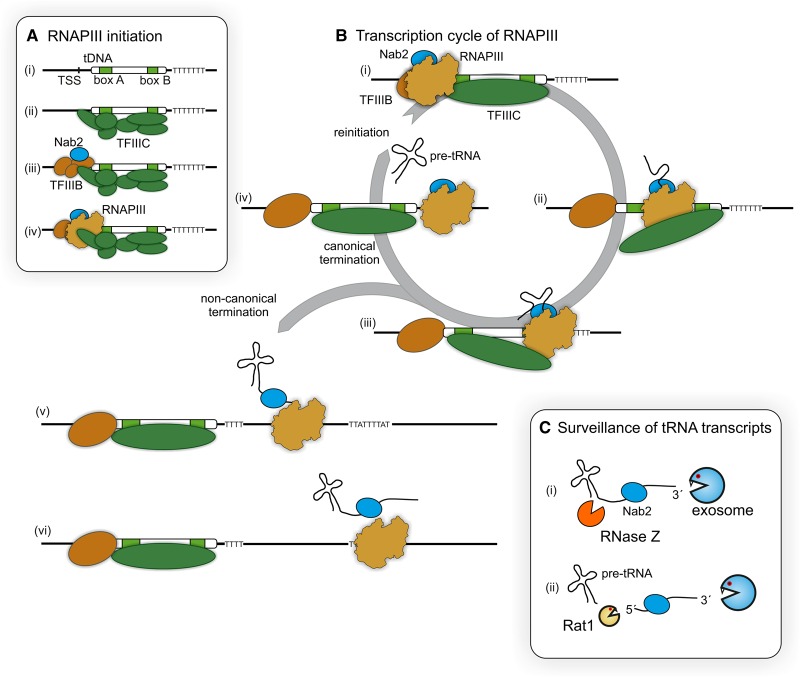
The RNAPIII transcription cycle. (**A**) RNAPIII transcription initiation on tRNA genes. (i and ii) The internal box A and B elements present within tRNA genes (tDNA) are bound by the multiprotein TFIIIC complex. (iii) This recruits the TFIIB complex and the RNA-binding protein Nab2 facilitates this interaction. (iv) RNAPIII is recruited and transcription initiates. (**B**) RNAPIII transcription cycle on tRNA genes. (i) During transcription elongation, Nab2 remains associated with RNAPIII and/or the nascent transcript. (ii and iii) TFIIIC remains associated with the tDNA, possibly because it is not simultaneously displaced from both the A and B boxes. (iv) In canonical termination, RNAPIII terminates following transcription of an oligo(U) tract. (v) In noncanonical termination, RNAPIII continues further downstream generating a readthrough product. (vi) This typically terminates in a U-rich region. (**C**) Pre-tRNA surveillance factors. (i) Readthrough transcripts are cleaved at the 3′-end of the mature tRNA, liberating a tRNA extension fragment, or can be trimmed on the 3′-end by the exosome complex. (ii) Released fragment of tRNA extension can be 5′ degraded by the 5′-exonuclease Rat1 (Xrn2 in humans) or 3′ degraded by the exosome complex. Nab2 may also participate in surveillance of 3′ extended pre-tRNAs.

Our understanding of events preceding and accompanying RNAPIII transcription initiation was largely derived from *in vitro* studies, but is supported by recent genome-wide analyses. TFIIIC recognizes and binds the internal promoter elements [7] and subsequently recruits TFIIIB upstream of the transcription start site (TSS) (Figure 1A) [8]. TFIIIB has been demonstrated to interact with Rpc34 during RNAPIII recruitment to DNA [9]. Events following transcription initiation are less well characterized (Figure 1B). Recent analyses in yeast revealed surprisingly uneven distribution of RNAPIII along individual transcription units [10,11]. In particular, a strong 5′-peak of RNAPIII occupancy was observed on all tRNA genes at the position of box A. This was interpreted as reflecting slow clearance from the initiation site, perhaps reflecting a delay in the dissociation of the polymerase from the transcription factor(s). A second peak of RNAPIII occupancy corresponded to the location of the B box, indicating that transcription through this transcription factor-binding site is also slowed. Gene occupancy by TFIIIC was reported to be lower than that of TFIIIB and RNAPIII, suggesting its displacement by transcription [12]. However, a different analysis found that TFIIIC was stably bound to the DNA template during transcription [13]. The recent data make it likely that TFIIIC is indeed associated in some way with the A and B boxes during transcription, resulting in the observed slowed elongation of RNAPIII. One possibility is that the elongating polymerase displaces TFIIIC from the DNA at box A then box B, but not from both sites simultaneously, allowing its continual association with actively transcribed genes (Figure 1B).

## RNAPIII termination and polymerase recycling

The best characterized transcription termination signal for RNAPIII consists of a tract of A residues on the template DNA strand. Minimum lengths are reported to be A_4_ for human and A_5_ or A_6_ for yeast [14–16], although recent analyses indicate that *in vivo* termination is significantly more effective with A_7_–A_8_ [11]. During termination, the weak base-pairing interactions between oligo(dA) in the template strand and oligo(U) in the nascent transcript act as a principal destabilizing signal. However, the nontemplate strand oligo(dT) tract also promotes polymerase pausing, formation of the pretermination complex and transcript release [17].

Studies in both yeast and human cells indicate a substantial level of RNAPIII transcription beyond the canonical oligo(U) terminator [11,18,19], with readthrough transcripts preferentially terminating at U-rich tract ≥50 nt from the 3′-ends of tRNAs [11,19]. In both budding and fission yeast, the readthrough transcripts are targeted for degradation by the exosome nuclease complex, preventing their accumulation to high levels [11,20]. In addition, the RNA-binding protein Nab2 is implicated in RNAPIII transcription [21], pre-tRNA quality control [22] and the degradation of readthrough products [11]. Nab2 interacts directly with RNAPIII and TFIIIB at the 5′-end of tRNA genes [21] and may couple transcription initiation with subsequent surveillance activities (Figure 1C).

*In vitro* analyses indicate that efficient RNAPIII transcription is promoted by frequent reinitiation following termination [23]. Transcription reinitiation efficiency may be linked to canonical termination of RNAPIII, since tRNA genes with the strongest termination readthrough generally showed lower transcription rates [11]. Noncanonical termination, at downstream sites, may reduce the reinitiation efficiency due to the loss of physical proximity between the terminating RNAPIII and transcription factors (Figure 1B).

## RNAPIII transcription is regulated in response to environmental signals

The internal promoter elements and TATA box, where present, are the only known *cis*-acting elements for RNAPIII transcripts. This strongly suggests regulatory mechanisms that are significantly simpler than for RNAPII. In yeast, the RNAPIII repressor Maf1 is the only known general regulatory factor [24], whereas mammalian RNAPIII is additionally regulated by Myc, p53 and retinoblastoma.

**Figure 2.**
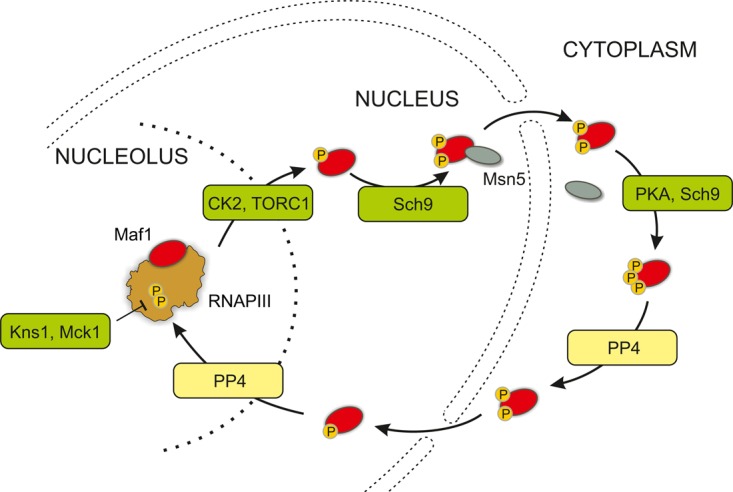
Regulated steps in RNAPIII transcription. The activity of the major transcription repressor Maf1 is regulated by cycles of phosphorylation and dephosphorylation, coupled with nuclear-cytoplasmic transport. These allow the integration RNPIII activity with nutrient availability and stress. See text for further details.

The activity of Maf1 is regulated by phosphorylation [25] (Figure 2), with dephosphorylated Maf1 binding to elongating RNAPIII and blocking subsequent transcription reinitiation [26]. In actively growing cells, Maf1 undergoes constant cycles of phosphorylation and dephosphorylation. Nuclear Maf1 can be phosphorylated by TORC1 [27–29], or the TOR-dependent kinase Sch9 [30], as well as CK2 [31]. Maf1-P is exported by the karyopherin Msn5 to the cytoplasm [25] where PKA and Sch9 kinases can also phosphorylate Maf1 [30,32]. Maf1-P is dephosphorylated by protein phosphatase 4 (PP4) [33] and imported into the nucleus, where it is active in RNAPIII repression. PP4 can also dephosphorylate Maf1-P in the nucleus. Nutrient deprivation blocks TORC1 and Sch9 activity, favoring Maf1-P dephosphorylation, nuclear import and tRNA repression (reviewed in ref. [34]). This two-component regulation, through phosphorylation and localization, allows for precise control of RNAPIII activity in response to environmental signals, including carbon source, nutrient availability and various stresses.[Fig BST-2016-0062F1]

A separate regulatory pathway involves direct phosphorylation of RNAPIII subunit, Rpc53, which is inhibitory for transcription. Kns1, a member of the LAMMER/Cdc family, acts as a priming kinase for phosphorylation by Mck1, a member of the GSK-3 family [35]. Deletion of both kinases has stronger effect than loss of either one. These kinases are downstream effectors of the TORC1 signaling pathway and also act to repress ribosome and tRNA synthesis in response to nutrient limitations.

Transcription of the 5S pre-rRNA may also be subject to feedback regulation, since the zinc-finger nucleotide-binding domain of transcription factor TFIIIA can bind both the *RDN5*-specific C box and the mature 5S rRNA (reviewed in ref. [36]). 5S rRNA is synthesized in excess of the other, RNAPI-transcribed, rRNAs and is found as a 7S RNP in complex with ribosomal proteins Rpl5 and Rpl11. In human cells, this RNP accumulates rapidly following stress-induced inhibition of ribosome synthesis and induces p53 stabilization and consequent cell-cycle arrest [37–39].

Little is known about transcriptional regulation of other RNAPIII transcribed genes, but it may be anticipated that the expression of these essential RNA species will be closely matched to cellular demands.

## A ‘housekeeping’ subset of the tRNA genes

The characterized tRNA transcription regulation pathways initially appeared to operate in a general manner across all genes. However, recent studies in yeast and human cells identified subsets of tRNA genes that are less responsive to transcription regulation [11,40]. Chromatin immunoprecipitation and sequencing on human cell lines revealed that, while most tRNA genes were strongly repressed by serum starvation, 49 active loci did not show significant changes [40]. These genes also showed only modest sensitivity to RNAi-mediated depletion of Maf1. Interestingly, most tRNA isotypes were represented within these genes. Analyses in the yeast *Saccharomyces cerevisiae* used UV cross-linking and analysis of cDNAs to identify nascent RNA associated with transcribing RNAPIII [11]. As in human cells, a subset of tRNA genes was less responsive to both environmental stresses (carbon source and temperature) and regulation by Maf1. This group contained acceptors for almost every amino acid. Additionally, measurements of growth yield in the tRNA gene deletion collection showed that identical tRNA gene copies contribute differentially [41]. Taken together, these studies strongly suggest the existence of a subset of ‘housekeeping’ tRNA genes that sustain basal cellular activities under adverse conditions. These tRNA genes are both less repressed under repressive condition and less up-regulated when the RNAPIII repressor Maf1 is absent. This raises the question of what features confer this variability between tRNA genes, particularly within closely related multigene families?

Many potential mechanisms might differentially regulate transcription of the same tRNA from different gene loci. Within tRNA families, the mature tRNAs share a common sequence, but the flanking sequences are different. Fragments of these flanking regions are transcribed as the 5′ leader and 3′ trailer of pre-tRNA, and are removed during the tRNA maturation process by a combination of endonuclease cleavage and exonuclease trimming (reviewed in ref. [42]). RNAPIII initiates within the 5′ upstream region of the gene, suggesting that the 5′ flanking sequence might be an important determinant in tuning tRNA transcription rates. Comparison of TSS with the 5′-end of the mature tRNA revealed that the TSS is variable on a subset of genes, most likely due to the sequence of the 5′ flanking region [11]. While preparing this review we reanalyzed the TSS of each tRNA gene (using data from ref. [11]) and found that 98% of tRNA transcripts starts with purines and 84% with an adenine residue. This indicates a strong sequence preference of RNAPIII, in agreement with previous biochemical data [43]. We speculate that the flanking regions of the ‘housekeeping’ tRNA genes are particularly favorable for recognition by the transcription machinery, and that this plays an important role in maintaining the basal transcription of this subset of tRNAs.

A potential distinctive feature of human housekeeping tRNAs could be chromatin modification patterns (reviewed by ref. [44]), since the regulation of tRNA genes in human cells is influenced by chromatin-modifying enzymes [45]. In contrast to yeast cells, where all tRNA genes are occupied by RNAPIII, in human cells only a subset of tRNA loci are in an active state [46]. Several features have been linked to establishment and maintenance of the transcriptionally active state, including binding by TFIIIC, TFIIIB, RNAPIII [47] and often by Myc [46]. tRNA genes that are located close to actively transcribed protein-coding genes, which carry histone modifications characteristic of euchromatin, may be present in a chromatin environment that promotes RNAPIII transcription. Conversely, the positioning of tRNA genes close to inactive RNAPII genes may suppress expression. It therefore seems possible that the housekeeping tRNA genes will show a characteristic chromatin modification pattern, but this has yet to be addressed.

## Nontranscriptional control of tRNA levels

Multiple routes have evolved to control mature tRNA levels, including changes in the gene copy numbers. Across all protein-coding genes in any genome, some codons are used more frequent than others — and each organism shows a characteristic bias in codon usage. The numbers of tRNA isoacceptor isotypes expressed in each organism are expected to match requirements for tRNAs in translation (see ref. [48] and references therein). For example, the yeast genome contains 275 tRNA genes with tRNA isoacceptor gene copy numbers that range from 1, recognizing rare anticodons (e.g. Ser^CGA^), to 16 each for tRNA^Gly(GCC)^ and tRNA^Asp(GTC)^ [49]. These numbers are even greater for the human genome, which contains some 610 tRNA genes, including 38 genes encoding tRNA^Cys(GCA)^. In most organisms, codon usage frequency is closely correlated with the number of tRNA genes, particularly for highly transcribed genes such as ribosomal protein genes [15].

The production of mature tRNAs can also be modulated by availability of the processing machinery. Following transcription, pre-tRNA processing generates the mature 5′- and 3′-termini, the 3′ CCA tail is added and tRNAs are exported to the cytoplasm. While the presence of intron-containing pre-tRNAs is conserved throughout eukaryotes, the cellular localization of the splicing event is not. In vertebrates, pre-tRNA splicing is nuclear, whereas the yeast SEN (splicing endonuclease) complex is, surprisingly, located on the cytoplasmic surface of mitochondria [50].

The early steps in pre-tRNA processing involve a dynamic interplay between maturation factors that may be affected by growth conditions [51]. The relative activities of the 3′ exonuclease Rex1 and the La protein (Lhp1 in yeast), which binds and protects the 3′ terminal oligo(U), affect pre-tRNA fates [52]. In *maf1Δ* cells growing under repressive conditions, levels of intron-containing pre-tRNAs were elevated [53], perhaps due to saturation of the export machinery for intron-containing pre-tRNAs [54]. These observations indicate that tRNA maturation is potentially controlled in response to growth conditions.

Mature tRNAs are both abundant (15% of total RNA) and very stable (life times of up to 72 h in vertebrates), with functional, charged tRNAs being protected by the highly abundant elongation factor 1A (eEF1A) [55,56]. The abundance of many classes of RNA is at least partly determined by turnover rates, but there is little evidence for the regulated turnover of stable/functional, mature tRNAs. In tRNAs that have failed to be correctly modified, both precursor and mature forms are targeted for degradation. Pre-tRNA^iMet^ lacking m^1^A58 modification is degraded in the nucleus by the exosome 3′ → 5′ nuclease complex, acting together with the TRAMP nuclear oligoadenylation complex [57–59]. In some cases, this degradation is triggered by extension of the CCA tail, probably reflecting mispairing in the acceptor stem [60]. Surprisingly, the effects of exosome inhibition indicate that a very substantial fraction of all pre-tRNA transcripts is removed as a result of competition between maturation and degradation factors [61,62]. In contrast, mature but unstable tRNAs are cleared by 5′ → 3′ exonucleases, nuclear Rat1 (Xrn2 in humans) and cytoplasmic Xrn1 [55,63,64].

In addition, the availability of tRNAs for translation can also be controlled, by regulating levels of tRNA charging, nuclear reimport of uncharged tRNA and the removal and re-addition of the CCA tails [65,66].

## tRNA (dys-)regulation in cancer

In oncogenically transformed cells, tRNAs are frequently substantially overexpressed, perhaps to support increased growth rates [67].

RNAPIII is regulated by well-known oncogenes and suppressor pathways including TORC1 (described above), Ras/Erk, Myc, p53 and Rb [68]. In untransformed cells, p53 and Rb repress RNAPIII transcription though direct interaction with TFIIIB. Binding of either p53 or Rb impairs TFIIIB binding to TFIIIC and therefore inhibits recruitment of RNAPIII [69,70]. Oncogenic transformation is often coupled with mutation or inactivation of those proteins. Consistently, overexpression of p53 or Rb represses RNAPIII transcription, whereas cells lacking p53 are characterized by increased RNAPIII activity.

The Ras family of conserved G-proteins regulates cell growth, proliferation, survival and differentiation. In many cancer cells, proliferation is driven by point mutations in Ras that lock it into a constitutively active state. Active Ras/Erk phosphorylates the TFIIIB component Brf1 and stimulates TFIIIB recruitment to the DNA template [71]. Another major oncogene, Myc, also binds TFIIIB and directly activates RNAPIII transcription [46]. Elevated expression levels of TFIIIC were found in ovarian carcinomas and fibroblasts transformed with DNA tumor viruses [72]. Moreover, overexpression of only the initiator tRNA^iMet^ is sufficient to drive transformation in a variety of cell types [73,74].

Comparison of tRNA levels in cancer cell lines with proliferating or differentiating cell lines identified two distinct subsets of tRNAs; one was specifically induced in proliferating cells (proliferation-induced), whereas the other was preferentially expressed during differentiation (differentiation-induced) [75]. Strikingly, the anticodon specificity of proliferation-induced tRNA correlates with the codon bias of genes that are up-regulated in proliferating or cancer cells. In breast cancer cells, higher levels of specific tRNAs allow for higher translation of genes containing complementary codons [76]. Notably, a direct link was shown between increased expression of tRNA^Glu(UUC)^ and *EXOSC2*, which encodes a component of the human exosome. Conversely, tRNA-derived fragments were reported to suppress breast cancer progression by displacing the RNA-binding protein YBX1 from oncogenic transcripts, resulting in their destabilization [77].

## Conclusions

**Figure 3.**
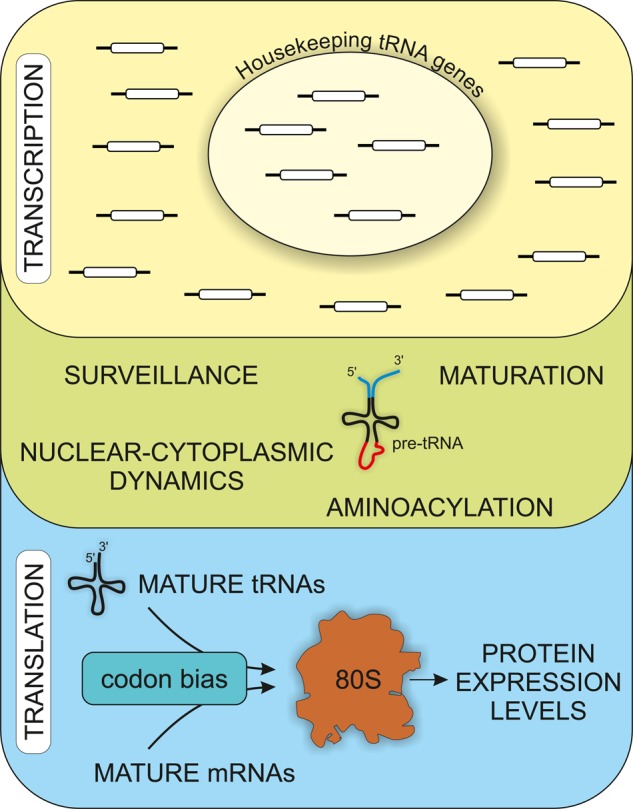
Changes in tRNA synthesis and maturation can affect protein production. The rate of pre-tRNA synthesis can be altered by changes in transcription rate, with a subset of ‘housekeeping’ tRNAs showing limited responses to environmental stress. In addition, tRNA abundance can be affected by changes in pre-tRNA processing, nuclear export and reimport, or surveillance activities. Different mRNAs show distinct patterns of codon bias, so changes in the relative tRNA abundances can alter protein expression levels.

For many years, tRNAs were viewed as passive factors whose sole function was to deliver a continual supply of aminoacids to the ribosomes. However, recent analyses are changing this view (Figure 3). Increased understanding of the *in vivo* kinetics, and differential regulation, of RNAPIII transcription has emerged from genome-wide analysis of nascent transcripts. It has become clear that tRNA abundance is modulated at multiple steps during synthesis and maturation. Moreover, individual tRNA species can be differentially regulated. In both yeast and human cells, a basal subset of tRNA genes show limited responses to both environmental changes and the major cellular repressor of tRNA synthesis. Changes in tRNA levels are important for cell proliferation and cancer progression, with increased abundance of specific tRNAs leading to enhanced translation of mRNAs with cognate codons. Understanding the mechanisms underpinning alterations in tRNA metabolism is therefore likely to be an important topic for future research.

## Abbreviations

PKA, protein kinase A; PP4, protein phosphatase 4; RNAPIII, RNA polymerase III; SEN, splicing endonuclease; snRNA, small nuclear RNA; SRP, signal recognition particle; TBP, TATA-binding protein; TF, transcription factor; TORC, TOR complex; TSS, transcription start site.

## Funding

This work was supported a grant from the Polish Ministry of Science and Higher Education Mobility Plus program to T.W.T. [1069/MOB/2013/0] and a Wellcome Trust Fellowship [077248] to D.T. Work in the Wellcome Trust Centre for Cell Biology is supported by Wellcome Trust core funding [092076].
